# Annual Report of the Department of Health of Buffalo

**Published:** 1887-11

**Authors:** 


### *ANNUAL REPORT OF THE DEPARTMENT OF HEALTH OF BUFFALO.*

For the first time in the history of our city, a report of the transactions of the Health Department has been published in pamphlet form. It may seem strange to many of our readers that such a report was never presented in former years. The labor devolving on Dr. Briggs has been enormous: to compel the registration of vital statistics, to provide a competent corps of assistants to enforce the health ordinances, and in a manner to educate both the people and the city officials up to the idea that preservation and promotion of the public health were really matters of as great importance as the lighting of streets or the introduction of natural gas.

Even now the Health Department has no adequate quarters for the transaction of business and the preservation of its records. The Health office is located in a corner of the City Clerk's office, indexes and records are filed away upon the floor, and the communications of the Health Officer are carefully stored away in a band-box; while park commissioners, assessors and even the coroners have offices of their own. Considering the disadvantages under which the Health Physician and his assistants have labored, we are surprised that so thorough and exhaustive a report has been compiled.

The Health Physician states in his report that the vital statistics show a larger birth-rate than any other city in the country. There was an *increase* in the number of births amounting to 696, marriages, 154; and a *decrease* in the number of deaths amounting to thirty-seven, as compared with the statistics of 1885.

Dr. W. H. Thornton contributes a report on the sanitary condition of the public schools, in which he shows that while the buildings recently constructed are in a fair sanitary condition, the older school-houses are in a deplorable state.

The City Chemist reports that of 267 samples of milk submitted to him for analysis, forty-nine or eighteen and thirty-five-hundredths per cent. were adulterated.

Of thirty-eight wells, the waters of which were analyzed, twenty-eight were condemned as impure, seven were passed, although the water was sufficiently hard to warrant condemnation under ordinary circumstances, and only three were found to contain wholesome drinking water.

Physicians will find the tables giving the death-rate from various classes of diseases for the six years from 1881 to 1886 inclusive, an interesting study, as they show how greatly particular classes of diseases vary in different years. On the whole, there has been a steady decline in the mortality from zymotic affections. There is also a report from Dr. F. R. Campbell on the "Relation of Locality to Mortality in Buffalo." This paper, which required an immense amount of statistical research, will be of great interest to the local physician. The tables are so arranged as to show the mortality from all causes, and from various zymotic diseases by wards, during the past six years. The various conditions affecting the mortality rate are also given. Taking the average mortality for six years, we learn from this table that the death-rate in the Ninth Ward from diarrhœal diseases and diphtheria has been one and one-tenth and fifty-eight-hundredths per 1,000 respectively, while in the Fifth Ward the death-rate from these diseases was four and fifty-two-hundredths and one and eighty-six-hundredths respectively. The First Ward has had uniformly the highest death-rate from diphtheria, excepting in 1882 and 1883, averaging for the six years two and thirty-eight-hundredths per 1,000—nearly double the average for the entire city.

Dr. Edward Clark contributes a valuable paper on "The Milk Supply of Cities," and the City Chemist an article on "The Water Supply of Buffalo," showing that we are provided with purer water than the great majority of large cities.

It is to be regretted that this report was not printed earlier in the year. Whether this delay was due to the negligence of the Health Officer or the public printer, we do not know, but it is certainly to be hoped that future reports will be published before they come to be classed among the antiquities of the city.

---

THE Buffalo Maternity Hospital, under the professional care of our editorial confreres, Drs. Lothrop and Frederick, has met with deserved success in this the second year of its work. It aims to provide a safe retreat for pregnant women, in which to pass their confinement and puerperal convalescence, under skillful obstetrical attendance, and with the most painstaking care and nursing. The institution meets a want long felt in this city, providing from among our ablest physicians an attending and consulting staff, which commands the endorsement and support of the profession here and elsewhere. Its success in the treatment of the puerperal period, under the principles of modern antisepsis, confirms the confidence we have always reposed in their application to puerperal women. We commend this institution and its work to our numerous friends, assuring them that it deserves the confidence of the profession at large, both in its professional work,and in its honorable administrative and financial management.

---

We desire to direct attention to the advertisement in this number of the Journal, of Wampole's Extract of Malt and Compound Syrup of Hypophosphites. These remedies have been largely used in our practice for several months, and the confidence, at first limited in their efficacy, has been augmented until they are now found among the reliable agents in our armamentarium medicum. The extract of malt combines the diastatic principle of the malt with the tonic qualities contained in porter, and makes an excellent remedy in feeble digestion, especially in cases which fail to digest the starchy foods.

The Compound Syrup of Hypophosphites is similar to Fellows', and much cheaper and equally reliable. We commend these new preparations to the profession, assuring them of their reliability.

---

Dr. F. A. Harrington, of this city, has been appointed Assistant to the Chair of Materia Medica and Therapeutics in the Medical Department of Niagara University. Dr. Harrington is a thoroughly educated man, being a graduate of both the academic and medical departments of Harvard University, and was for one year resident physician in the Boston Lying-in Hospital. In 1886, he received the degree of A. M. *cum laude* from his *alma mater.* Dr. Harrington is to be congratulated on having his abilities recognized by the profession so early after entering upon his practice in Buffalo, and the Medical Faculty of Niagara University has done well in thus filling the subordinate position with a young practitioner of such thorough preliminary training.

